# A database of life history parameters for Pacific coral reef fish

**DOI:** 10.1038/s41597-025-05788-x

**Published:** 2025-08-14

**Authors:** Anela K. Akiona, Brian J. Zgliczynski, Marisa M. Agarwal, Beverly J. French, Nathaniel Hanna Holloway, Katie A. Lubarsky, McClaran E. Shirley, Christopher J. Sullivan, Stuart A. Sandin

**Affiliations:** Scripps Institution of Oceanography, UC San Diego, San Diego, California 92093 USA

**Keywords:** Marine biology, Marine biology, Tropical ecology, Community ecology

## Abstract

Length-weight parameters assist in the estimation of a fish’s biomass based upon assessment of length, providing value to many scientific and management applications. Some fish species have many sets of parameters published, while those less commonly studied may have no available information, making it challenging to decide which values to use. To address this, we present a database of quality-controlled length-weight parameters for 1,308 Pacific coral reef fish species from 87 families. The species included are commonly observed in visual censuses of shallow (10 m depth) forereef habitats of the central Pacific. These data were collated from the literature, and were selected based on study sample size, representation of the species’ total length range, and data confidence. The most speciose families, wrasses (Labridae) and damselfishes (Pomacentridae), were most common in our database, though a large proportion of these species remain data-poor. In total, this database serves as a valuable tool for the academic community as well as for partners and managers desiring to learn more about coral reef fish assemblages.

## Background & Summary

Length and weight are fundamental biological traits for all living organisms. Among many animal taxa, these two characteristics are linked through allometric scaling, which describes how changes in one dimension affect changes in another as an organism grows. First coined by Huxley & Tessier^1^, allometry can provide a simplification of how animals adjust their shape and physiology as they increase in size, with linked changes in body dimensions affecting an individual’s mobility, energy requirements, and survival strategies^1,2^. Length-weight relationships (LWR) are frequently modeled through the power function formula

1 $$W=a{L}^{b}$$

where *W* represents weight, *L* is length, and *a* and *b* are species-specific constants. This model has broad applications in ecology because it enables scientists to predict various life-history traits and examine how they relate to each other, making it a powerful tool in understanding population dynamics and interspecies interactions^3^.

In fisheries ecology, LWR are particularly important as they allow fisheries scientists to estimate the weight of a fish based on its length, enabling rapid assessments of fish health and population dynamics without intensive or extractive sampling efforts^4–7^. To maintain precision of weight estimation, capturing reliable parameterization of LWR is essential. Notably, LWR are species-specific and can differ based on factors such as body morphology, geographic region, and environmental conditions^3,8–10^, which makes parameter selection an important step of data analysis. Fisheries managers rely on the insights provided by LWR to establish sustainable fishing practices^7,11^, set regulations^12^, and monitor the health of aquatic ecosystems^13,14^, particularly when non-invasive methodologies are utilized.

Among the most common applications of LWR are fish assemblage descriptions based upon underwater visual census (UVC). UVC is a fisheries-independent tool for assessing and monitoring fish assemblages, and generates important data metrics such as fish abundance, biomass, size-structure, biodiversity, and trophic structure. UVC encompasses several different survey techniques (e.g., belt transect surveys, stationary point count (SPC), etc.) in which divers visually identify and estimate the number and size of fish within a given area, often while using SCUBA^15–19^. Compared to fisheries-dependent studies, UVC is a non-destructive approach which allows for rapid data collection and provides a snapshot view of fish communities at each survey site. On coral reefs, UVC can provide a cost-effective way to quickly survey diverse fish assemblages^18^. The derived data yield useful ecological insights into the fish assemblages, as well as the potential effects of various disturbances on those assemblages. For example, data on the abundance and size structure of fish populations between fished and unfished systems can be used to evaluate the effectiveness of marine protected areas^4,7,20,21^. Further, when repeated over time, UVCs can show how coral reef fish communities change in response to events such as coral bleaching, habitat modification, marine protection, and the introduction of invasive species^22,23^.

While UVC methods excel at capturing the diversity of diurnally active, non-cryptic coral reef fishes, transforming these observational data into meaningful biomass estimates presents significant challenges. The conversion of fish counts and size estimates into biomass relies fundamentally on LWR; however, the extraordinary biodiversity of coral reef ecosystems, with thousands of fish species across the Pacific Ocean alone, has resulted in substantial gaps in available LWR parameters for many species. Researchers often address these data gaps by applying parameters from related species or from different geographic regions, a practice that can introduce significant bias into biomass estimates^3,24^. While Bayesian approaches have been developed to estimate missing parameters based on taxonomic relationships, these methods may not fully capture the complex ecological and evolutionary factors that influence fish morphology and condition^25^.

With increasing availability and collection of life history parameters, it is inevitable that global databases (e.g., FishBase^26^) would expand to include numerous parameters for one species, particularly species with global distributions. As one example, *Cephalopholis argus*, a common grouper, has ten different sets of *a-b* values to choose from in the FishBase database as of publication^26^. While some of these life history parameters are flagged as suspect, the process of choosing the best parameter for any particular species from a given region still elicits many questions, especially with multiple options that all seem equally valid^27^. How does an individual researcher prioritize among the many options in a reproducible way?

Here we present an empirically derived, quality-controlled database of length-weight parameters for commonly observed coral reef fishes from the tropical Pacific region. We have created an openly available, regionally expansive list of known length-weight parameters for regional partners (e.g., practitioners, resource managers) and the scientific community. Similar efforts to compile and serve species-specific data have proved successful in other scientific disciplines^28–30^. We intend for this database to serve as a powerful resource for the Pacific region, compiling the best available information on fish life history parameters. There are other global fish databases, such as FishBase^26,31^, which aim to provide comprehensive species-level data for finfish. We do not seek to replace these efforts, but rather to build from them to provide complementary, expert-level curation based on the underlying life history data. To this end, we provide a curated list of length-weight parameters to use for analyses. Additionally, we provide R code for a function that uses length-weight parameters to calculate biomass and generate summary figures from UVC data. One of our motivations in creating this database is to lessen the burden on individual researchers and managers by conducting this curation for the Pacific region. A secondary outcome is increased standardization and comparison between monitoring programs, which are essential for worldwide summaries of the status of coral reefs and their fisheries, such as the Global Coral Reef Monitoring Network^32^.

The taxa targeted for inclusion in this database were those identified as the most commonly observed by UVC on forereef habitats of the tropical Pacific. Based upon UVC data collected across 13 Pacific island nations, a list of 1,308 unique fish species was identified representing 87 families. While the most observed families – and thus most represented species in this database – were wrasses (Labridae) and damselfish (Pomacentridae), they were also the taxa which utilized parameters from congeners the most; 126 of 203 Labrids and 93 of 148 Pomacentrids did not have species-specific data (Table S1). The selected length-weight parameters for the taxa included in this database were derived from 91 source publications. These publications spanned 13 biogeographic realms as defined by Costello *et al*.^33^, with varying degrees of geographic resolution. While the compiled taxon list was generated from surveys conducted within the Pacific, not all the selected length-weight parameters were derived from studies based in this region. For example, the most suitable set of length-weight parameters for the red lionfish (*Pterois volitans)* – a species native to the Indo-Pacific – came from research conducted in the tropical Western Atlantic, where the species is considered non-native^34^. Despite the potential variation in body shape or allometric growth rates between populations of a species across geographic ranges, some length-weight parameters were necessarily selected from outside of the Pacific basin when they represented the most robust data available.

At present, most resource managers and coral reef ecologists rely on scientific manuscripts, technical reports, and online resources such as FishBase^26,31^ to access length-weight parameters that support individual life history assessments. The compilation of length-weight parameters can be time consuming and can lack data standardization or quality control across data sources. In most cases, each database includes data from a variety of published and online sources – each being unique to an individual or group – making it difficult to identify the source of the original length-weight parameters or validate the resulting estimates of fish biomass included in each project. Furthermore, in some cases length-weight parameters are selected without consideration for data quality or potential sources of error.

Previous efforts to collect and compile length-weight parameters for coral reef fishes have provided an invaluable source of life history information. These often span a range of scientific objectives; some studies collect length-weight parameters across a range of species, while others focus on collecting parameters on a single species^11^ or a group of species^35,36^. In some cases, studies rely on empirical data from targeted collections or fisheries-dependent observations, while other efforts use statistical modeling approaches (e.g., Bayesian estimates) to estimate length-weight parameters. The approaches by which resource managers and members of the scientific community interpret and utilize these data can be variable. For example, in the case of ecological or fisheries-independent studies, length-weight parameters are used to estimate the biomass of fishes observed during UVC, and hastily chosen length-weight parameters can result in over- or under- estimated biomass values^3,37^. It is therefore important to evaluate the sources of length-weight data and choose parameters based on criteria that best support individual project objectives.

Another challenge in the application of LWR is the potential for geographic conditions influencing parameter estimates. For species that realize large amounts of geographic variability in LWR, there can be cause for concern in application of empirical parameters without proper validation or consideration of geographic origin. In some cases, significant variation in LWR have been documented across geographies of the Pacific Ocean, driven by regional oceanographic conditions, biogeographic patterns, and varying anthropogenic pressures^38,39^. Further, fish populations of the same species may exhibit markedly different length-weight relationships between the geographic regions due to differences in productivity regimes, water temperature, and food availability^40–42^.

The spatial variability in length-weight relationships underscores the critical importance of using locally or regionally appropriate parameters when estimating fish biomass. Failure to account for regional effects can lead to systematic biases in biomass estimates, potentially compromising the accuracy or precision of ecological assessments, fisheries management decisions, and cross-system comparisons. Therefore, researchers must carefully consider the geographic origin of length-weight parameters and their applicability to the system of interest, particularly when conducting large-scale comparative studies across ocean basins. Furthermore, the use of a standardized, citable set of length-weight parameters such as those presented herein allows for more reliable comparisons of coral reef fish biomass across studies.

In this study we identify five criteria that we recommend colleagues should consider when selecting length-weight parameters: (1) Regional differences in length-weight parameters of a species – the life history characteristics of a species are influenced by biotic factors and the biogeographic ranges of most coral reef fishes span across environmental gradients^40–42^; (2) Sample size – efforts to collect length-weight parameters from targeted species can be variable and the number of individual samples included in a study can range from one to hundreds or even thousands of individuals; however, because length-weight parameters are based on scaling relationships and linear regression, smaller sample sizes will have less statistical confidence; (3) Size range across samples – since fish growth rates change with ontogeny, it is important to consider sampling efforts where individuals are collected across the entire range of size classes for a targeted species^24,37^; (4) Parameters from species vs. from congeners (or similar taxon) – based on available data, the length-weight parameters for a targeted species may be limited or lacking. In these cases, it may be advantageous to select length-weight parameters of a congener or from a species with similar morphometrics if the selection criteria are better met by that species; (5) Empirical data vs. Bayesian estimates – Bayesian point estimates have been found to differ significantly from species-specific, empirically derived LWR, and the posterior distributions of the Bayesian predictions often have large uncertainties around point estimates^43^. Given that the Bayesian predictions are based on only four different body morphologies, we advocate for the use of robust species-specific LWR when available, or a suitable congener when it is not.

As the field of coral reef ecology advances there is a concerted effort to make use of length-weight parameters to increase our understanding of the structure and function of fishes. We must also recognize opportunities to build upon previous efforts and fill gaps in available life history information, such as leveraging the marine aquarium trade. We found that there was an overrepresentation of length-weight parameters for large-bodied and popular fishery target species. In contrast, some of the most ecologically important (e.g., reef sharks) and numerically abundant (e.g., anthias – *Pseudanthias* spp. and chromis – *Pychnochromis* spp.) groups of fishes are lacking length-weight parameters. Other important groups such a coral-associated (e.g., hawkfish – Cirrhitidae) or obligate corallivores (nested within the lower-carnivore group, e.g., butterflyfish – Chaetodontidae) are underrepresented in the literature and there is a need to fill data gaps. Moving forward, we encourage colleagues to contribute to efforts of LWR estimation and share data whenever possible. By working collaboratively, we can continue advancing our understanding of these valuable ecosystems.

## Methods

To create a database of nearshore, diurnally active coral reef species for the tropical Pacific, we extracted a taxon list based on the results of UVC surveys conducted by the Sandin Lab to characterize coral reef communities from 13 countries across the region from 2005-2023, which generally targeted forereefs at 10 m depth. Once all scientific names from the compiled taxa were confirmed as valid in the World Register of Marine Species (WoRMS) (last accessed October 2023), this list served as the basis for the length-weight database.

Length-weight parameters were gathered from the literature, primarily through searches on Google Scholar or Web of Science. All length-weight parameter data sources are listed in the ‘Citation Information’ tab within the main data file and cited in this paper^34–36,44–132^. FishBase was used to supplement this search and it also served as the primary reference for biological information on each species, including but not limited to biogeographic range, maximum length and weight, diet, and length-to-length ratios. When information was not readily available on FishBase, fish reference books^133–135^ and online literature searches for manuscripts and technical reports provided species and life-history information. As most UVC length estimates are total length (TL), we calculated a conversion from the length measurement of the source publication (if it was done in standard or fork length) to TL using either length-to-length ratios provided on FishBase or through manual calculations.

If there were multiple sources for the length-weight parameters, we developed a process for selecting the most accurate parameters based on the following criteria, listed in order of priority: study sample size, size range of fish measured in the study, and data confidence. For example, if two studies reported the same sample size but one study recorded length values covering 20% of the species length range while the other study recorded values for 60%, the parameters from the study with the greater length range would be selected. Data confidence (r or R^2^) was used as a tie breaker if all other criteria were equal. Parameters were chosen from the Pacific as much as possible, though studies from outside the Pacific were used when they represented the most robust data available. It is important to note that while much thought and consideration has been given to the parameters included in the database based on the criteria outlined above, our search was not comprehensive and therefore did not necessarily consider all published parameters for every species.

When a species did not have any reliable length-weight parameters available, parameters from a congener which met the above criteria were used. Congeners were selected based on similarity of body morphology and maximum length. In cases where genus or family level parameters were not available in the literature, length-weight parameters were chosen from a species we felt was representative of that taxon, rather than trying to average species-level parameters together^83^. Given the diversity of fish morphology, we did not feel confident providing these for all families and genera. It is also worth noting that what is representative of a genus or family is regionally specific, and we encourage users of this database to choose these parameters based on what makes sense for their study region.

### Trophic designations

We chose to include broad trophic categories for the interested reader, recognizing that many reef fishes have flexible diets which are context-dependent and shift with ontogeny^136^. The classifications were selected to provide general guidance regarding the trophic position of the species and are not intended to represent the current state of knowledge regarding species-specific diets. We define the classifications as follows: (1) *Herbivore/detritivore*: the herbivore/detritivore group includes species that derive most of their food through foraging on non-planktonic primary producers. As such, this group includes species that feed primarily on turf algae, coralline algae, and other macroalgae. Further, the group includes species that forage among benthic algae yet derive the bulk of their nutrition from small invertebrates, detritus, or microbes found within the algae; (2) *Planktivore*: the planktivore group includes species that feed primarily on planktonic organisms. The group principally includes species that feed on zooplankton, though many species also forage opportunistically on phytoplankton, and the group includes species foraging on both allochthonous and autochthonous sources of plankton; (3) *Lower-carnivore*: the lower-carnivore group includes benthic invertivores and species that feed primarily on small fishes, but do not have as large of a possible prey base as top predators; (4) *Top predator*: we use a working definition of ‘top predator’ based upon the extent of the potential prey base, following Sandin *et al*.^137^, which are principally species of groupers (Serranidae), jacks (Carangidae), and snappers (Lutjanidae), among others; and (5) *Sharks*: this grouping includes all shark species of the elasmobranch fishes. We have separated this grouping from top predators, as not all sharks are considered top or apex predators^138^. While trophic designations for this group are particularly controversial and often size-dependent, sharks are often studied or noted for their distinct ecological roles on reefs, and as such, we include them as a separate category.

## Data Records

The complete database is publicly available on the University of California San Diego Library Digital Collections website (10.6075/J02Z15WT)^139^. The primary data file, ‘Pacific_LW_parameters_V1.xlsx’, contains a quality-controlled, curated set of parameters for each species, which are located on the ‘Length-Weight Parameter Table’ tab. Parameters were selected from the literature based on sample size, representation of species length range, and data confidence. In cases where suitable parameters were not available, a representative species with robust parameters was chosen as a substitute. Detailed citation information, including the biogeographic realm, can be found in the ‘Citation Information’ tab. Updated versions of this database will be made available as new parameters are published and subsequently reviewed by our group.

## Technical Validation

The database of life history parameters for Pacific coral reef fishes was curated as described above in methods. All compiled data were inspected to ensure that the data were contained within, and any parameters that could not be traced to a primary source were excluded. Values were input verbatim to the source document, excepting length-to-length ratios calculated manually, which were rounded to the nearest 0.01. After final data compilation, we used the Taxon Match Tool (https://www.marinespecies.org/tutorial_taxonmatch.php) to ensure that taxonomy was consistent with the World Register of Marine Species (WoRMS)^140^.

## Usage Notes

The dataset is hosted via the University of California San Diego Library Digital Collections website (10.6075/J02Z15WT)^139^. The UC San Diego Research Data Collections contain research data generated by campus researchers, as supported by the Research Data Curation Program, and are publicly available and searchable to anyone for educational and research use. Notably, the Excel file containing the database additionally includes references to all original sources that were used to compile the length-weight parameters. We therefore encourage users to reference these sources along with this data descriptor when using the selected parameters. We further note that the curated database is an evolving data product, as generation of new length-weight parameters continues, along with further data curation and addition to the database. Given this, we encourage users to access the latest versions of the database files, which will be updated as new, dated versions in the UC San Diego Library Digital Collections. Subsequent versions of the database will be marked with their data of deposition, along with the date at which data curation ceased for a particular version. Each version will have a unique DOI, and for reproducibility we encourage users to cite the DOI of the specific database version used.

## Supplementary information

Supplementary Table S1

## Data Availability

Though there was no custom code used to generate this database, code to utilize the length-weight parameters presented herein to calculate fish biomass from UVC can be found on Github at https://github.com/aakiona/fish-summarizer/.
